# Aspirin for primary prevention of cardiovascular disease

**DOI:** 10.1186/s12959-015-0068-7

**Published:** 2015-12-04

**Authors:** Jobert Richie N. Nansseu, Jean Jacques N. Noubiap

**Affiliations:** Department of Public Health, Faculty of Medicine and Biomedical Sciences of the University of Yaoundé I, PO Box 1364, Yaoundé, Cameroon; Sickle Cell Disease Unit, Mother and Child Centre of the Chantal Biya Foundation, Yaoundé, Cameroon; Department of Medicine, Groote Schuur Hospital and University of Cape Town, Cape Town, South Africa; Medical Diagnostic Centre, Yaoundé, Cameroon

**Keywords:** Aspirin, Cardiovascular disease, Primary prevention

## Abstract

Although aspirin has a well-established role in preventing adverse events in patients with known cardiovascular disease (CVD), its benefit in patients without a history of CVD remains under scrutiny. Current data have provided insight into the risks of aspirin use, particularly bleeding, compared with its benefits in primary CVD prevention. Although aspirin is inexpensive and widely available, especially in developing countries, there is lack of evidence that the benefits outweigh the adverse events with continuous aspirin use in primary CVD prevention. Therefore, the decision to initiate aspirin therapy should be an individual clinical judgment that weighs the absolute benefit in reducing the risk of a first cardiovascular event against the absolute risk of major bleeding, and tailored to the patient’s CVD risk. This risk must be calculated, based on accurate and cost-benefit locally developed risk assessment tools, the most discriminating threshold be identified. Additionally, patients preferences should be taken into account when making the decision to initiate aspirin therapy in primary prevention of CVD or not. Physicians should continuously be trained to calculate their patients CVD risk, and concomitant strategies be emphasized.

## Introduction

Cardiovascular disease (CVD), of which coronary heart disease (CHD) and stroke are the prevailing components, is by far the leading cause of death in most developed countries and is rapidly becoming the leading cause of death in the world [1]. Indeed, the World Health Organization estimates that annual global mortality due to CVD will approach 25 million by 2030, of which about 80 % will occur in developing countries [2]. These alarming and potentially avoidable trends result mainly from major increases in cigarette smoking, obesity, and physical inactivity in both developed and developing countries [1].

Because of the prominent role of thrombosis in the pathogenesis of CVD, anti-thrombotic agents have been tested for the prevention of CVD. The anti-thrombotic properties of aspirin were first described in 1971 [3], and the hypothesis that aspirin could efficiently prevent CVD was subsequently raised and investigated. In fact, aspirin therapy can reduce the risk of major cardiovascular events such as heart attack and stroke [4]. Aspirin is widely available and very inexpensive as well, and its utilization in the prevention of CVD would be highly profitable to the global population, in particular those without prior CVD, and specifically those living in low-income countries.

Although there is body of evidence that aspirin is beneficial in secondary CVD prevention [5, 6], findings from clinical trials and meta-analyses on aspirin benefit in primary CVD prevention are not homogenous. When tested for primary prevention in clinical trials of predominantly low risk individuals, aspirin has been shown to decrease the rate of CVD events but at a near-equivalent risk of increased bleeding [6–9], limiting its use only in individuals at elevated risk for a cardiovascular event, and thereby withholding aspirin from lower risk patients who represent the majority of the primary prevention population and in whom a large proportion of cardiovascular events occur [10]. Consequently, guidelines and other expert opinions differ substantially in their recommendations for aspirin use in primary CVD prevention, reflecting the uncertainty of a precise risk/benefit ratio in this context.

Limiting aspirin use to only high-risk individuals negates the opportunity to prevent a significant number of cardiovascular events, many of which present as unheralded myocardial infarction (MI) or sudden cardiac death [11], especially in developing countries where alternatives to aspirin could be unaffordable by the large majority of primary CVD prevention populations [12–14]. The present review revisits published knowledge on the safety and efficacy of aspirin use in primary prevention of CVD and presents current practical recommendations.

## Review

### The theoretical physiological effects of aspirin in cardiovascular disease prevention

For a cardiovascular event to occur, the fibrous cap of an atheromatous lesion must rupture. This plaque rupture exposes highly pro-thrombotic material leading to thrombosis [15–17]. Therefore, the prevention of thrombus formation or the prevention of propagation of thrombus once it exists will consequently prevent evolution into full vascular occlusion, hence the utility of anti-thrombotic agents.

Aspirin is a non-selective cyclo-oxygenase (COX) inhibitor. It blocks the production of thromboxane in platelets (by acetylation of COX in platelets) thereby inhibiting their aggregation, but it also blocks the synthesis of prostaglandins in the vascular wall that, in health, causes vasorelaxation, maintains renal function, and reduces adhesion of platelets to the vessel wall [3, 18]. Furthermore, aspirin may be beneficial for atherosclerosis not only because of its antiplatelet action, but also by showing a direct effect on the atheroma plaque. Indeed, Redondo et al. have undoubtedly demonstrated that aspirin increases the stability of the atheroma plaque (or significantly inhibits plaque growth), this mediated by an increase in TGF-β secretion [19, 20]. These properties of aspirin have consequently brought to explore the benefits of this drug in CVD prevention. However, the use of aspirin, especially in the long term, is not without some harmful effects that can even become fatal.

### Adverse events with aspirin

The major drawback to continuous aspirin therapy is the risk of bleeding. The Antithrombotic Trialists’ Collaboration (ATTC) meta-analysis revealed that aspirin use increased the risk of major gastrointestinal (GI) and other extracranial bleeding by about 50 % (0.10 %/year vs. 0.07 %/year (risk ratio (RR) 1.54, 95 % confidence interval (CI):1.30–1.82; *p* <0.0001) [6]. Aspirin also increases the risk of hemorrhagic stroke. A meta-analysis of 16 placebo-controlled randomized controlled trials (RCTs), comprising a total of 55,462 patients, showed that treatment with aspirin was associated with a relative risk of hemorrhagic stroke of 1.84 (p < 0.001) [21]. Likewise, The ATTC reported a statistically significant 22 % increased incidence of hemorrhagic stroke in patients on antiplatelet treatment [22]. Moreover, De Beradis et al. [23] in comparison with ATTC estimates, found a 5-time higher incidence of major bleeding events with an overall incidence rate of hemorrhagic events of 5.58 (95 % CI: 5.39–5.77) per 1000 person-years for aspirin users and 3.60 (95 % CI: 3.48–3.72) per 1000 person-years for those without aspirin use (incidence ratio rate (IRR) 1.55, 95 % CI: 1.48–1.63). In particular, the use of aspirin was associated with an excess risk of gastrointestinal (IRR 1.55, 95 % CI: 1.46–1.65) and intracranial bleeding (IRR 1.54, 95 % CI: 1.43–1.67) [23]. In the recent Japanese Primary Prevention Project (JPPP) [24], aspirin significantly increased the risk of extracranial hemorrhage requiring transfusion or hospitalization (0.86, 95 % CI: 0.67–1.11 for aspirin vs. 0.51, 95 % CI: 0.37–0.72 for no aspirin; hazard ratio (HR) 1.85, 95 % CI: 1.22–2.81; *p* = 0.004).

Several other studies have also shown that the risk of bleeding is increased with aspirin therapy [25–31]. It has been emphasized that this risk is higher in the primary prevention than in the secondary prevention population, but this difference is more than compensated by the lower baseline risk in primary prevention population [26]. Likewise, the risk of bleeding is higher in individuals at higher cardiovascular risk over 10 years when compared with the low-risk population [32]. Furthermore, some other factors likely to dreadfully increase the risk of bleeding have been identified: increased age, previous hospitalization for GI problems, helicobacter pylori infection, male sex, diabetes, hypertension, previous cardiovascular problems, alcohol use, concurrent use of nonsteroidal anti-inflammatory drugs (NSAIDs) or concomitant use of either antiplatelet agents or oral anticoagulants [6, 23, 33, 34]. These risk factors are common in the general population and therefore, the individual patient’s risk of bleeding needs to be taken into consideration when deciding to put him/her on continuous aspirin therapy, especially in low-income settings where there may be lack of enough means and competence to handle such related harmful side effects [14].

Some bleeding risk scores have been developed, especially in anticoagulated atrial fibrillation (AF) populations, such as the HEMORR_2_HAGES score, the HAS-BLED score, and the less practical ATRIA score. We learn from Lip G’s review that the HAS-BLED score has been shown to outperform the other risk scores in predicting clinically relevant bleeding in multiple ‘real-world’ and trial cohorts [35]. Additionally, the HAS-BLED score was the only tested score to have a significant (and good, c-statistic 0.75) predictive value for intracranial haemorrhage [36]. The acronym HAS-BLED represents each of the bleeding risk factors and assigns 1 point for the presence of each of the following: hypertension (uncontrolled systolic blood pressure >160 mmHg), abnormal renal and/or liver function, previous stroke, bleeding history or predisposition, labile international normalized ratios, elderly, and concomitant drugs and/or alcohol excess [37]. The HAS-BLED score ranges from 0 to 9, with scores of ≥ 3 indicating high risk of bleeding, for which caution and regular review of the anticoagulated AF patient are recommended [35, 38]. In this regard, the applicability of such a score should be undertaken in primary prevention of CVD with aspirin, and patients at high risk of bleeding should not be prescribed aspirin in primary prevention of CVD.

Moreover, the standard dosage of aspirin (325 mg/day) is significantly associated with a higher risk of gastrointestinal bleeding (including fatal bleeds) than are low-doses (75–100 mg/day) [39–41], though this risk is estimated to be twice as high as with no aspirin [42]. Some papers have pointed-out that an increased consumption of proton pump inhibitors (PPIs) might be associated with a significant reduction in hospitalizations for gastrointestinal events during continuous aspirin therapy [23, 43], but the cost-effectiveness of PPIs associated with aspirin use, specifically in resource-poor environments, needs a thorough clarification. Earnshaw et al. [44] demonstrated that the addition of a PPI to aspirin is cost-effective only for men at increased risk for gastrointestinal bleeding. On the other hand, there have been some claims that using PPIs might increase the risk of cardiovascular events in patients treated with antiplatelet agents [45]. However, it is questionable if the PPI used (omeprazole) was the cause of this increased risk of adverse cardiovascular events, or if omeprazole use was merely a mark of a more severe patient (omeprazole users tend to be older, more fragile and with more comorbidities, all factors increasing mortality). Other authors have proposed that aspirin could be substituted by another anti-thrombotic agent to reduce CVD risk while eliminating the bleeding risk, but two prospective clinical trials demonstrated for instance that the combination of aspirin and esomeprazole was superior to clopidogrel for the prevention of recurrent ulcer bleeding [46, 47].

Aspirin use is also associated with an increased risk of dyspepsia, chronic kidney disease and renal dialysis, and age-related macular degeneration [48]. There is some suggestion that low-dose aspirin (50 mg daily) might not slow carotid atheroma plaque growth [49]. Consequently, any effect of aspirin on cardiovascular events needs to be balanced against the potential for harm. In primary CVD prevention where the risk of developing atherothrombotic events is generally low, it is therefore highly relevant to estimate the individual baseline risk of such events and carefully balance this against the risk of harmful events related to therapy.

### Assessing the baseline risk in primary prevention of cardiovascular disease

Commonly-used tools to assess baseline risk are the Framingham coronary heart disease risk score [32], the American College of Cardiology/American Heart Association (AHA/ACC) Task Force risk equations [50], and the European Society of Cardiology ESC’s SCORE (Systematic Coronary Risk Evaluation) [51]. The Framingham risk score (FRS) utilizes gender, age, total cholesterol, high-density lipoprotein cholesterol, systolic blood pressure, use of blood pressure lowering medications, and smoking status to predict the 10-year risk of developing a coronary event (composite of MI and coronary death). Individuals are categorized as low (<10 %), moderate (10 to 20 %), or high (>20 %) risk [32]. Moreover, the SCORE system estimates the 10-year risk of a fatal atherosclerotic event: individuals are considered at low risk with a SCORE < 1 %, at moderate risk with a SCORE ≥ 1 % and <5 %, at high risk with a SCORE ≥ 5 % and <10 %, and at very high risk with a SCORE ≥ 10 % [51]. The risk of total fatal and nonfatal events is higher than that of fatal events only. At a 5 % risk of fatal events, the total event risk is approximately 15 %. This 3-fold multiplier is somewhat smaller in the elderly, in whom a first event is more likely to be fatal [51].

Additionally, the Coronary artery calcium (CAC) score has recently been tested to guide aspirin utilization for primary prevention of CVD. In fact, one can read from Miedema et al. [11] that CAC score is a highly specific marker of the atherosclerotic plaque burden in the coronary arteries, and there is a nearly 10-fold higher risk of CHD events in patients with substantially elevated CAC. The same authors report that a CAC score of zero has been shown to be a powerful predictor of a favorable prognosis (low risk of CHD, CVD and all-cause mortality), even in the presence of traditional risk factors [11, 52]. Furthermore, an analysis of 44,052 asymptomatic people showed that individuals with no cardiovascular risk factors but elevated CAC had higher mortality rates than individuals with multiple risk factors but zero CAC [53]. Although CAC may be a promising risk assessment and reclassification tool, how this test should be used in routine clinical practice remains unclear, added to its relatively high cost ($100).

Another tool that has been used is the ankle brachial index (ABI) which is the ratio of systolic pressure at the ankle to the arm. It is used in the diagnosis of peripheral artery disease affecting the legs. A low ABI is associated with concomitant coronary and cerebrovascular disease [54], and, in healthy individuals, with an increased risk of future vascular events, independently of cardiovascular risk factors [55]. An ABI of 0.90 or less is often used as the cut point for identifying individuals at high risk of peripheral atherosclerosis. [55]. In this regard, Fowkes et al. [28] hypothesized that, because the ABI is a simple, inexpensive, noninvasive test, it could be used in population screening programs (for example in primary care settings or in the community) to identify a novel subgroup potentially amenable to preventive treatments.

However, as most of these aforementioned tools were developed in Caucasian populations, their application in other populations or ethnic groups is therefore problematic. In this regard, the INTERHEART risk score was developed based on data from 52 countries around the world [56]. Yusuf et al. [56] showed that the INTERHEART risk score, based on nine easily measureable and potentially modifiable risk factors, is associated with more than 90 % of the likelihood to accurately predict an acute MI, these results being consistent across all geographic regions and ethnic groups of the world, men and women, and young and old. More recently, McGorrian et al. [57] modified the INTERHEART risk score into the INTERHEART Modifiable Risk Score (IHMRS), of which four risk score models were derived. Of these, the ‘non-laboratory-based’ score [57], which does not include any lab-based measures of lipid profile, can be used in primary care settings especially in resource-poor countries where there is lack of laboratories [14].

Consequently, more observational studies as well as direct randomized comparisons between all these commonly-used tools are urgently warranted to define which is the most reliable and cost-effective one to be used for primary CVD risk assessment, particularly in low-income settings [58]. Besides, adoption of continuous aspirin use for primary CVD prevention must be underpinned by robust scientific evidence.

### Published evidence for or against a beneficial effect of aspirin in primary prevention of cardiovascular disease

In 2009, the ATTC reviewed the benefit-risk profile of low-dose aspirin for primary CVD prevention in a comprehensive meta-analysis of 6 primary prevention trials that included nearly 95,000 men and women randomly assigned to aspirin at doses of 75–500 mg per day or placebo [6]. Aspirin was associated with a 12 % proportional reduction in serious vascular events compared with no aspirin (annual event rate 0.51 % for aspirin vs. 0.57 % for no aspirin, *p* <0.0001), mainly due to a reduction in first nonfatal MI (0.18 % vs. 0.23 % per year; *p* <0.0001), and the proportional reductions were similar for women and men [6]. Conversely, aspirin therapy did not seem to have an effect on stroke occurrence, and it was noticed just a small protective effect with regard to mortality, given 0 to 6 fewer deaths per 1000 persons treated over 10 years. However, as early mentioned, the downside was that aspirin increased major gastrointestinal and extracranial bleeding [6]. Since bleeding risk appeared to be strongly related to the ischemic risk, the benefit of aspirin was judged to be overshadowed by the bleeding hazard.

More recent meta-analyses suggested beneficial effects for aspirin in the primary prevention of cardiovascular events [8, 9, 30, 31, 59, 60]. Indeed, Bartolucci et al. [59] reported in their meta-analysis that aspirin significantly decreased the risk of total cardiovascular events (OR 0.87, 95 % CI: 0.80–0.93; *p* <0.001) and nonfatal MI (OR 0.81, 95 % CI: 0.67–0.99; *p* = 0.042), compared with no aspirin, but there was a non-significant trend for decreased risk of stroke, cardiovascular mortality, and all-cause mortality. In the meta-analysis performed by Raju et al. [60], primary prevention with aspirin was associated with a reduction in all-cause mortality (RR 0.94, 95 % CI: 0.88–1.00), MI (composite of fatal and nonfatal; RR, 0.83, 95 % CI: 0.69–1.00), ischemic stroke (RR 0.86 95 % CI: 0.75–0.98), and the composite of MI, stroke, and cardiovascular death (RR 0.88, 95 % CI: 0.83–0.94) in comparison with nonuse of aspirin. In another meta-analysis conducted by Seshasai et al. [9], aspirin association with a significant reduction in the risk of cardiovascular events (OR, 0.90, 95 % CI: 0.85–0.96), was primarily accounted for by a large reduction in the risk of nonfatal MI (OR 0.80, 95 % CI: 0.67–0.96). No effect on fatal MI was observed, but a modest non-significant reduction was apparent for all-cause mortality [9].

Moreover, the Multi-Ethnic Study of Atherosclerosis that involved 4229 participants who were not on aspirin at baseline and were free of diabetes mellitus revealed that participants with significant plaque in their arteries (i.e., CAC score ≥100) were estimated to be 2 to 4 times more prone to prevent a heart attack with aspirin use than to have a major bleed secondary to aspirin [27]. On the contrary, participants with no calcified plaque (CAC score = 0) were estimated to be 2 to 4 times more likely to suffer a major bleed from aspirin use than to prevent a heart attack with aspirin [27]. In the just-published JPPP that included 14,464 patients aged 60–85 years and presenting with either hypertension, dyslipidemia, or diabetes mellitus, low-dose aspirin (100 mg/day) significantly reduced incidence of nonfatal MI (0.30, 95 % CI: 0.19-0.47 for aspirin vs. 0.58, 95 % CI: 0.42–0.81 for no aspirin; HR 0.53, 95 % CI: 0.31–0.91; *p* = 0.02) [24]. Likewise, it was noticed a significant reduction in the incidence of transient ischemic attack (0.26, 95 % CI: 0.16–0.42 for aspirin vs. 0.49, 95 % CI: 0.35–0.69 for no aspirin; HR 0.57, 95 % CI: 0.32–0.99; *p* = 0.04) [24].

However, this study was terminated early, based on likely futility. In fact, it appeared on the whole that aspirin was unlikely to show a clinically important benefit in the overall population included in the study [24]. The drug did not significantly reduce the risk of the composite outcome of cardiovascular death, nonfatal stroke, and nonfatal MI, and the risk of bleeding significantly increased with aspirin [24]. In accordance with these findings, a systematic review conducted by Jones et al. [61] showed no differences between aspirin and placebo groups for total and vascular mortality, MI, and stroke in patients with asymptomatic peripheral artery disease. In the same line, Brighton et al. [62] showed no reduction in recurrent thromboembolic events with aspirin use of 100 mg/day even though there was a clear reduction in the occurrence of other serious cardiovascular events by 34 % (*p* = 0.01) compared to the placebo group. In addition, no differences in bleeding or other serious adverse events were observed [62]. The Aspirin for Asymptomatic Atherosclerosis trial did not show a significant reduction in fatal or non-fatal coronary event, stroke or revascularization among participants without clinical CVD identified with a low ABI and receiving aspirin in comparison with placebo [28].

Overall, in large-scale trials of primary prevention in men and women without established CVD, and subsequent meta-analyses, aspirin significantly reduced the risk of a first MI, but this was less the case for stroke or cardiovascular death. But despite the apparent benefits of aspirin therapy for primary prevention of CVD, these benefits may be outweighed by the risk of major bleeding (see Table 1) [6, 8, 9, 30, 31]. Indeed, a recent extensive systematic review of aspirin in primary prevention concluded that “there is a fine balance between benefits and risks from regular aspirin use in primary prevention of cardiovascular disease” [31].

**Table 1:** Summarizing published evidence for or against a beneficial effect of aspirin in primary prevention of cardiovascular disease

Study	Beneficial effects	No effect/harmful effects
The Antithrombotic Trialists’ Collaboration (ATTC) meta-analysis [6]	- A 12 % proportional reduction in serious vascular events, mainly due to a reduction in first nonfatal myocardial infarction	- No effect on stroke occurrence
		- Increase in the risk of major gastrointestinal and other extracranial bleeding by about 50 %
	- Small protective effect on mortality	- Increased incidence of hemorrhagic stroke (22 %)
Seshasai et al. [9]	- Significant decrease in the risk of cardiovascular events (notably nonfatal myocardial infarction)	No effect on fatal myocardial infarction
	- Modest non-significant reduction in all-cause mortality	
He et al. [21]	Absolute risk reduction in myocardial infarction and ischemic stroke	Increased risk of hemorrhagic stroke
De Beradis et al. [23]		High incidence of major bleeding events with an overall incidence rate of hemorrhagic events (gastrointestinal and intracranial bleeding episodes)
The Japanese Primary Prevention Project (JPPP) [24]	- Significant reduction in the incidence of nonfatal myocardial infarction	- No significant diminution in the risk of the composite outcome of cardiovascular death, nonfatal stroke, and nonfatal myocardial infarction
	- Significant reduction in the incidence of transient ischemic attack	
		- Increase in the risk of extracranial hemorrhage requiring transfusion or hospitalization
The Multi-Ethnic Study of Atherosclerosis [27]	Estimated 2–4 fold increased likelihood to prevent a heart attack with aspirin use than to have a major bleed secondary to aspirin in participants with significant plaque in their arteries (i.e., CAC score ≥100).	Estimated 2–4 times increased probability to suffer a major bleed from aspirin use than to prevent a heart attack with aspirin in participants with no calcified plaque (CAC score = 0)
The Aspirin for Asymptomatic Atherosclerosis trial [28]		No significant reduction in fatal or non-fatal coronary event, stroke or revascularization among participants without clinical cardiovascular disease identified with a low ankle brachial index and receiving aspirin in comparison with placebo
Bartolucci et al. [54]	Significant decrease in the risk of total cardiovascular events and nonfatal myocardial infarction	No effect on the reduction in the risk of stroke, cardiovascular mortality, and all-cause mortality
Raju et al. [55]	Reduction in all-cause mortality, myocardial infarction (composite of fatal and nonfatal), ischemic stroke, and the composite of myocardial infarction, stroke, and cardiovascular death	Increase in the risk of hemorrhagic stroke, major bleeding, and gastrointestinal bleeding
Jones et al. [56]		No differences between aspirin and placebo groups for total and vascular mortality, myocardial infarction, and stroke in patients with asymptomatic peripheral artery disease
Brighton et al. [57]	- Reduction in the occurrence of serious cardiovascular events (by 34 %) other than thromboembolic events	No reduction in recurrent thromboembolic events (with aspirin use of 100 mg/day)
	- No differences in bleeding or other serious adverse events	

#### The specific case of diabetes patients

Individuals with diabetes have a 2 to 4 fold increased risk of developing cardiovascular events than those of the same age and sex without diabetes [63]. Haffner et al. [63] have shown that the risk of future CHD events is similar for both diabetes subjects without prior CHD and non-diabetes subjects with previous CHD. Besides, a meta-analysis by the ATTC suggested that diabetes, in addition to being an independent CVD risk factor, also increased the risk of extracranial hemorrhage or bleeding [6]. Conversely, Feigin et al. [64] reported a protective effect of diabetes on the incidence of subarachnoid hemorrhages, which they justified by figuring out the high risk of death from other causes in the population with diabetes and therefore the chance of developing such hemorrhages was smaller than in individuals without diabetes.

The Early Treatment Diabetic Retinopathy Study (ETDRS) [65] is the main study supporting the use of aspirin in primary prevention of diabetes patients. This multicenter, randomized clinical trial of aspirin versus placebo investigated the effects of aspirin (325 mg/day) in primary prevention on 3711 type 1 and type 2 diabetes patients aged 18–70 years old with various degrees of retinopathy, and showed that aspirin did not affect retinopathy progression, but was rather associated with a reduction in the incidence of fatal and nonfatal MI [65]. Consistent with these findings, the Physicians’ Health Study [66], a randomized double-blind placebo-controlled trial designed to determine whether aspirin decreased cardiovascular events in primary prevention in patients with diabetes, demonstrated a significant reduction in the risk of MI, though without conclusive evidence concerning stroke and total cardiovascular deaths.

Paradoxically, Simpson et al. [41] carried-out a meta-analysis to examine the effect of dose on the association between aspirin and cardiovascular outcomes using data from 21 studies with 17,522 diabetes patients, and found that aspirin use was not associated with a statistically significant difference in mortality risk. More recently, Sasso et al. [25] did not show a significant difference in the incidence of both fatal (*p* = 0.225) and nonfatal CV events (*p* = 0.573) between type 2 diabetes patients with nephropathy placed on aspirin vs. placebo. These findings are in keeping with other studies including the Japanese Primary Prevention of Atherosclerosis with Aspirin for Diabetes (JPAD) [29], the Prevention and Progression of Arterial Disease and Diabetes (POPADAD) trial [67], the Primary Prevention Project Trial [68] and the meta-analysis of seven randomized clinical trials regrouping 11,000 patients and undertaken by Butalia et al. [69]. On the whole, although aspirin may not necessarily increase the risk of bleeding in diabetes patients, recent available data suggest no benefit in terms of a reduction in vascular events.

### Prescribe aspirin in primary prevention of CVD: a challenging decision

Currently, the worthwhile net clinical benefit of giving aspirin to healthy individuals is made difficult to assess by the imprecision of estimates of benefits and risks, especially for rare events, such as intracranial hemorrhage, and by the difficulty of weighing ischemic versus bleeding events [70, 71]. In the setting of secondary cardiovascular prevention on the contrary, the benefit of treatment (saving major cardiovascular events) is clearly superior to the risk (inducing major bleeding) [6].

The American Heart Association (AHA), the American College of Chest Physicians (ACAP), and the European Society of Cardiology (ESC) have formulated recommendations for the use of low-dose aspirin for primary cardiovascular prevention with some of them being conflicting (see Table 2). For instance, while the ACAP guidelines suggests daily low-dose aspirin for persons age 50 years or older without symptomatic CVD, ESC guidelines argues that aspirin is “not recommeded in individuals without cardiovascular or cerebrovascular disease” because of the increased risk of major bleeding. Despite certain inconsistences, all guidelines are clear that aspirin is inappropriate for patients with aspirin intolerance and those at increased risk of gastrointestinal bleeding or hemorrhagic stroke, which is usually evident from a previous history of such conditions. These aspirin contraindications are relatively uncommon, however and happily, and the benefits of CVD risk reduction typically outweigh the bleeding risks for most patients at high CVD risk. It is worth noting that aspirin doses of 75–162 mg per day are typically better tolerated and are as effective as higher doses in conferring cardiovascular protection [72].

**Table 2:** Recommendations for low-dose aspirin therapy in cardiovascular disease primary prevention

Organization	Statement
American Heart Association/American Stroke Association [78, 79]	a) The use of aspirin for cardiovascular (including but not specific to stroke) prophylaxis is reasonable for people whose risk is sufficiently high (10-year risk >10 %) for the benefits to outweigh the risks associated with treatment.
	b) Aspirin can be useful for the prevention of a first stroke among women, including those with diabetes mellitus, whose risk is sufficiently high for the benefits to outweigh the risks associated with treatment.
	c) Aspirin might be considered for the prevention of a first stroke in people with chronic kidney disease (ie, estimated glomerular filtration rate <45 mL/min/1.73 m^2^). This recommendation does not apply to severe kidney disease (stage 4 or 5; estimated glomerular filtration rate <30 mL/min/1.73 m^2^).
	d) Aspirin is not useful for preventing a first stroke in low-risk individuals with or without diabetes.
	e) Use of aspirin for primary cardiovascular prevention is reasonable in diabetic patients whose 10-year risk of events is > 10 % (men age > 50 years and women age > 60 years with at least 1 additional risk factor: smoking, hypertension, dyslipidemia, albuminuria, or family history of premature cardiovascular events) and who are not at increased risk of bleeding (no history of gastrointestinal bleeding or peptic ulcer disease, no concurrent use of other medications that increase bleeding risk).
	f) Aspirin may be considered for diabetes patients at intermediate risk of cardiovascular events (younger patients with at least 1 risk factor, older patients with no risk factors, or patients with a 10-year risk of 5 to 10 %)
American College of Chest Physicians [32, 80]	Aspirin is recommended for the primary prevention of cardiovascular events in all patients aged 50 years or older with or without symptomatic cardiovascular disease.
Canadian Cardiovascular Society [81]	Aspirin is not recommended in men or women without evidence of manifest vascular disease.
European Society of Cardiology [82]	Aspirin is not recommended in individuals without cardiovascular or cerebrovascular disease.

Definitely, in primary CVD prevention, where the risk of developing atherothrombotic events for each individual patient is generally low, it is essential to estimate the individual baseline risk of such events and to carefully balance this against the side effects of therapy, in this case bleeding. The ability to prevent serious cardiovascular events and their associated sequelae with a single, inexpensive pill makes low-dose aspirin therapy a worth considering low cost option, specifically in impoverished areas. Therefore, tailoring aspirin therapy according to baseline risk has been proposed as the best practical guidance [11, 34, 70, 73]. As such, a threshold risk level should be defined, above which recommending aspirin is expected to produce more benefit than risk, and assessed through the most accurate and country-specific risk factor estimates. In this regard, the ESC working group on thrombosis has proposed a threshold at a risk of major cardiovascular events (death, MI and stroke) ≥ 2 per 100 patient-years [70].

Any decision to prescribe aspirin should be an “individual clinical judgment” that weighs the absolute benefit in reducing the risk of a first cardiovascular event against the absolute risk of major bleeding [73]. The higher the absolute risk of CVD, the greater the net benefit from aspirin. This final decision to initiate continuous aspirin therapy for primary prevention of CVD should be made by health care providers [73], especially Primary Care Physicians (PCPs) in resource-poor areas, and they must therefore be cognizant of their role in assessing the potential for benefit and subsequent risk of bleeding in individual patients prior to starting the treatment.

But mirroring previous findings, Fiscella et al. [74] figured out that the use of aspirin in primary prevention of CVD is hindered by the clinician uncertainty in determining relative benefits and harms to patients. In fact, they found that aspirin was recommended for only 34 and 42 % of eligible men and women, likely reflecting the complexity of assessing patient eligibility, including weighing benefits and harms in the context of risk calculations and age-based risk cutoffs [74]. In the absence of hand-based or online risk calculators, physicians often misjudge CVD global risk. It has therefore been suggested that incorporation of automated CVD risk assessments into electronic health records could improve clinicians’ estimation of risk and recommendation for prescribing [75]. But the implementation of such a measure in resource-limited zones is questionable, given that in most of these areas patients health records are not yet computerized and local risk assessment tools are lacking [14, 58]. In this regard, simple adapted tools must be developed and PCPs be continuously trained to use them.

Besides, engaging non-clinicians through expanded care teams and standing orders may also prove helpful in reducing the volume of decisions that rest solely with the clinician at the point of care [74]. Indeed, sharing care with team members can enhance the concordance between published guidelines, use of risk models and actual practice [76]. Additionally, the relevant information should be transferred to patients and, individual values and preferences be taken into account. Indeed, patient preferences may play a larger role, but consideration must also be given to clinical equivalence as many patients may be more willing to experience a bleeding event as opposed to suffering a heart attack [27]. It has been stated that providing patients with global CVD risk assessments seems to improve patient intent to initiate CVD prevention [77].

Lastly, therapeutic lifestyle changes should be emphasized or reinforced [73]. In this line, populations should be sensitized on healthy and life-saving attitudes (healthy diets, physical activity, and smoking cessation among others). Further, other life-saving drugs such as statins could be considered with aspirin as an adjunct, or gastrointestinal protection be given in association with aspirin. But these measures have shown controversial results and their implementation in resource-constraints settings must be guided by cost-effective evidence.

## Conclusion

The widespread and “appropriate” use of aspirin in prevention of CVD is particularly attractive because the drug is generally widely available and, more importantly for developing countries where CVD is becoming the leading cause of death, is extremely inexpensive. Although the benefit of aspirin treatment is clear in secondary CVD prevention, the evidence in primary prevention remains unclarified. In fact, the studies revisited point out an increased risk of major gastrointestinal, extracranial and intracranial bleeding with continuous aspirin therapy. Given this uncertainty about aspirin’s effects and the differences in how people may assess the beneficial and adverse consequences of aspirin use in primary CVD prevention, providers and patients should routinely discuss aspirin use within the context of an overall strategy for CVD prevention that is tailored to the patient’s CVD risk. This risk must be calculated, based on accurate and cost-benefit locally developed risk assessment tools, and adequate thresholds be defined.

## References

[1] Lim SS, Vos T, Flaxman AD, Danaei G, Shibuya K, Adair-Rohani H (2012). A comparative risk assessment of burden of disease and injury attributable to 67 risk factors and risk factor clusters in 21 regions, 1990–2010: a systematic analysis for the Global Burden of Disease Study 2010. Lancet.

[2] World Health Organization (2015). Atlas of heart disease and stroke.

[3] Miner J, Hoffhines A (2007). The discovery of aspirin’s antithrombotic effects. Tex Heart Inst J.

[4] Hayden M, Pignone M, Phillips C, Mulrow C (2002). Aspirin for the primary prevention of cardiovascular events: a summary of the evidence for the US Preventive Services Task Force. Ann Intern Med.

[5] Bhatt DL, Eagle KA, Ohman EM, Hirsch AT, Goto S, Mahoney EM (2010). Comparative determinants of 4-year cardiovascular event rates in stable outpatients at risk of or with atherothrombosis. JAMA.

[6] Baigent C, Blackwell L, Collins R, Emberson J, Godwin J, Antithrombotic Trialists’ (ATT) Collaboration (2009). Aspirin in the primary and secondary prevention of vascular disease: collaborative meta-analysis of individual participant data from randomised trials. Lancet.

[7] Berger JS, Roncaglioni MC, Avanzini F, Pangrazzi I, Tognoni G, Brown DL (2006). Aspirin for the primary prevention of cardiovascular events in women and men: a sex-specific meta-analysis of randomized controlled trials. JAMA.

[8] Berger JS, Lala A, Krantz MJ, Baker GS, Hiatt WR (2011). Aspirin for the prevention of cardiovascular events in patients without clinical cardiovascular disease: a meta-analysis of randomized trials. Am Heart J.

[9] Seshasai SR, Wijesuriya S, Sivakumaran R, Nethercott S, Erqou S, Sattar N (2012). Effect of aspirin on vascular and nonvascular outcomes: meta-analysis of randomized controlled trials. Arch Intern Med.

[10] Cooney MT, Dudina A, Whincup P, Capewell S, Menotti A, Jousilahti P (2009). Re-evaluating the Rose approach: comparative benefits of the population and high-risk preventive strategies. Eur J Cardiovasc Prev Rehabil.

[11] Miedema MD, Cohn JN, Garberich R, Henry J, Graham KJ, Henry TD (2012). Under-use of cardiovascular preventive pharmacotherapy in patients presenting with ST-elevation myocardial infarction. Am Heart J.

[12] Jingi AM, Noubiap JJ, Ewane Onana A, Nansseu JR, Wang B, Kingue S (2014). Access to diagnostic tests and essential medicines for cardiovascular diseases and diabetes care: cost, availability and affordability in the West Region of Cameroon. PLoS One.

[13] Mendis S, Fukino K, Cameron A, Laing R, Filipe A, Khatib O (2007). The availability and affordability of selected essential medicines for chronic diseases in six low- and middle-income countries. Bull World Health Organ.

[14] Mendis S, Al Bashir I, Dissanayake L, Varghese C, Fadhil I, Marhe E (2012). Gaps in capacity in primary care in low-resource settings for implementation of essential noncommunicable disease interventions. Int J Hypertens.

[15] Davies MJ, Thomas A (1984). Thrombosis and acute coronary artery lesions in sudden cardiac death. N Engl J Med.

[16] Davies MJ, Thomas AC (1985). Plaque fissuring—the cause of acute myocardial infarction, sudden ischaemic death, and crescendo angina. Br Heart J.

[17] Santos-Gallego CG, Picatoste B, Badimón JJ (2014). Pathophysiology of acute coronary syndrome. Curr Atheroscler Rep.

[18] Patrono C, Ciabattoni G, Patrignani P, Pugliese F, Filabozzi P, Catella F (1985). Clinical pharmacology of platelet cyclooxygenase inhibition. Circulation.

[19] Redondo S, Santos-Gallego CG, Ganado P, García M, Rico L, Del Rio M (2003). Acetylsalicylic acid inhibits cell proliferation by involving transforming growth factor-beta. Circulation.

[20] Redondo S, Santos-Gallego CG, Tejerina T (2007). TGF-beta1: a novel target for cardiovascular pharmacology. Cytokine Growth Factor Rev.

[21] He J, Whelton PK, Vu B, Klag MJ (1998). Aspirin and risk of hemorrhagic stroke: a meta-analysis of randomized controlled trials. JAMA.

[22] Antiplatelet Trialists’ Collaboration (2002). Collaborative meta-analysis of randomised trials of antiplatelet therapy for prevention of death, myocardialinfarction, and stroke in high risk patients. BMJ.

[23] De Berardis G, Lucisano G, D’Ettorre A, Pellegrini F, Lepore V, Tognoni G (2012). Association of aspirin use with major bleeding in patients with and without diabetes. JAMA.

[24] Ikeda Y, Shimada K, Teramoto T, Uchiyama S, Yamazaki T, Oikawa S (2014). Low-dose aspirin for primary prevention of cardiovascular events in Japanese patients 60 years or older with atherosclerotic risk factors: a randomized clinical trial. JAMA.

[25] Sasso FC, Marfella R, Pagano A, Porta G, Signoriello G, Lascar N, et al. Lack of effect of aspirin in primary CV prevention in type 2 diabetic patients with nephropathy: results from 8 years follow-up of NID-2 study. Acta Diabetol. 2014; [Epub ahead of print]. 10.1007/s00592-014-0623-x 25109286

[26] Lin KJ, De Caterina R, García Rodríguez LA (2014). Low-dose aspirin and upper gastrointestinal bleeding in primary versus secondary cardiovascular prevention: a population-based, nested case–control study. Circ Cardiovasc Qual Outcomes.

[27] Miedema MD, Duprez DA, Misialek JR, Blaha MJ, Nasir K, Silverman MG (2014). Use of coronary artery calcium testing to guide aspirin utilization for primary prevention: estimates from the multi-ethnic study of atherosclerosis. Circ Cardiovasc Qual Outcomes.

[28] Fowkes FG, Price JF, Stewart MC, Butcher I, Leng GC, Pell AC (2010). Aspirin for prevention of cardiovascular events in a general population screened for a low ankle brachial index: a randomized controlled trial. JAMA.

[29] Ogawa H, Nakayama M, Morimoto T, Uemura S, Kanauchi M, Doi N (2008). Low-dose aspirin for primary prevention of atherosclerotic events in patients with type 2 diabetes: a randomized controlled trial. JAMA.

[30] Sutcliffe P, Connock M, Gurung T, Freeman K, Johnson S, Ngianga-Bakwin K (2013). Aspirin in primary prevention of cardiovascular disease and cancer: a systematic review of the balance of evidence from reviews of randomized trials. PLoS One.

[31] Sutcliffe P, Connock M, Gurung T, Freeman K, Johnson S, Kandala NB (2013). Aspirin for prophylactic use in the primary prevention of cardiovascular disease and cancer: a systematic review and overview of reviews. Health Technol Assess.

[32] Vandvik PO, Lincoff AM, Gore JM, Gutterman DD, Sonnenberg FA, Alonso-Coello P (2012). Primary and secondary prevention of cardiovascular disease: antithrombotic therapy and prevention of thrombosis, 9th ed: American College of Chest Physicians Evidence-Based Clinical Practice Guidelines. Chest.

[33] Patrono C, Garcia Rodriguez LA, Landolfi R, Baigent C (2005). Low-dose aspirin for the prevention of atherothrombosis. N Engl J Med.

[34] Park K, Bavry AA (2013). Aspirin: its risks, benefits, and optimal use in preventing cardiovascular events. Cleve Clin J Med.

[35] Lip GY (2013). Stroke and bleeding risk assessment in atrial fibrillation: when, how, and why?. Eur Heart J.

[36] Apostolakis S, Lane DA, Guo Y, Buller H, Lip GY (2012). Performance of the HEMORR(2)HAGES, ATRIA, and HAS-BLED bleeding risk-prediction scores in patients with atrial fibrillation undergoing anticoagulation: the AMADEUS (evaluating the use of SR34006 compared to warfarin or acenocoumarol in patients with atrial fibrillation) study. J Am Coll Cardiol.

[37] Pisters R, Lane DA, Nieuwlaat R, de Vos CB, Crijns HJ, Lip GY (2010). A novel user-friendly score (HAS-BLED) to assess 1-year risk of major bleeding in patients with atrial fibrillation: the Euro Heart Survey. Chest.

[38] Lane DA, Lip GY (2012). Use of the CHA(2)DS(2)-VASc and HAS-BLED scores to aid decision making for thromboprophylaxis in nonvalvular atrial fibrillation. Circulation.

[39] Campbell CL, Smyth S, Montalescot G, Steinhubl SR (2007). Aspirin dose for the prevention of cardiovascular disease: a systematic review. JAMA.

[40] Mehta SR, Bassand JP, Chrolavicius S, Diaz R, Eikelboom JW, CURRENT-OASIS 7 Investigators (2010). Dose comparisons of clopidogrel and aspirin in acute coronary syndromes. N Engl J Med.

[41] Simpson SH, Gamble JM, Mereu L, Chambers T (2011). Effect of aspirin dose on mortality and cardiovascular events in people with diabetes: a meta-analysis. J Gen Intern Med.

[42] Weil J, Colin-Jones D, Langman M, Lawson D, Logan R, Murphy M (1995). Prophylactic aspirin and risk of peptic ulcer bleeding. BMJ.

[43] Russo P, Brutti C (2007). Proton pump inhibitors and hospital discharge rates for gastrointestinal events in Italy: a national ecological study. Clin Ther.

[44] Earnshaw SR, Scheiman J, Fendrick AM, McDade C, Pignone M (2011). Cost-utility of aspirin and proton pump inhibitors for primary prevention. Arch Intern Med.

[45] Charlot M, Grove EL, Hansen PR, Olesen JB, Ahlehoff O, Selmer C (2011). Proton pump inhibitor use and risk of adverse cardiovascular events in aspirin treated patients with first time myocardial infarction: nationwide propensity score matched study. BMJ.

[46] Lai KC, Chu KM, Hui WM, Wong BC, Hung WK, Loo CK (2006). Esomeprazole with aspirin versus clopidogrel for prevention of recurrent gastrointestinal ulcer complications. Clin Gastroenterol Hepatol.

[47] Chan FK, CHing JY, Hung LC, Wong VW, Leung VK, Kung NN (2005). Clopidogrel versus aspirin and esomeprazole to prevent recurrent ulcer bleeding. N Engl J Med.

[48] Cleland JG (2013). Is aspirin useful in primary prevention?. Eur Heart J.

[49] Ranke C, Hecker H, Creutzig A, Alexander K (1993). Dose-dependent effect of aspirin on carotid atherosclerosis. Circulation.

[50] Goff DC, Lloyd-Jones DM, Bennett G, Coady S, D’Agostino RB, Gibbons R (2013). ACC/AHA guideline on the assessment of cardiovascular risk: a report of the American College of Cardiology/American Heart Association Task Force on Practice Guidelines. J Am Coll Cardiol.

[51] Perk J, De Backer G, Gohlke H, Graham I, Reiner Z, Verschuren M (2012). The fifth joint task force of the european society of cardiology and other societies on cardiovascular disease prevention in clinical practice (constituted by representatives of nine societies and by invited experts). Eur Heart J.

[52] Amarenco P, Bogousslavsky J, Callahan A, Goldstein LB, Hennerici M, Rudolph AE (2006). High-dose atorvastatin after stroke or transient ischemic attack. N Engl J Med.

[53] Nasir K, Rubin J, Blaha MJ, Shaw LJ, Blankstein R, Rivera JJ (2012). Interplay of coronary artery calcification and traditional risk factors for the prediction of all-cause mortality in asymptomatic individuals. Circ Cardiovasc Imaging.

[54] Newman AB, Siscovick DS, Manolio TA, Polak J, Fried LP, Borhani NO (1993). Ankle-arm index as a marker of atherosclerosis in the cardiovascular health study. Cardiovascular Heart Study (CHS) Collaborative Research Group. Circulation.

[55] Collaboration ABI, Fowkes FG, Murray GD, Butcher I, Heald CL, Lee RJ (2008). Ankle brachial index combined with Framingham risk score to predict cardiovascular events and mortality: a meta-analysis. JAMA.

[56] Yusuf S, Hawken S, Ounpuu S, Dans T, Avezum A, Lanas F (2004). Effect of potentially modifiable risk factors associated with myocardial infarction in 52 countries (the INTERHEART study): case–control study. Lancet.

[57] McGorrian C, Yusuf S, Islam S, Jung H, Rangarajan S, Avezum A (2011). Estimating modifiable coronary heart disease risk in multiple regions of the world: the INTERHEART Modifiable Risk Score. Eur Heart J.

[58] Noubiap JJ, Nansseu JR. Are the current recommendations for the use of aspirin in primary prevention of cardiovascular disease applicable in low-income countries? Vasc Health Risk Manag. 2015; In press. 10.2147/VHRM.S87398 PMC4556041 26345154

[59] Bartolucci AA, Tendera M, Howard G (2011). Meta-analysis of multiple primary prevention trials of cardiovascular events using aspirin. Am J Cardiol.

[60] Raju N, Sobieraj-Teague M, Hirsh J, O’Donnell M, Eikelboom J (2011). Effect of aspirin on mortality in the primary prevention of cardiovascular disease. Am J Med.

[61] Jones WS, Schmit KM, Vemulapalli S, Subherwal S, Patel MR, Hasselblad V (2013). Treatment strategies for patients with peripheral artery disease [Internet].

[62] Brighton TA, Eikelboom JW, Mann K, Mister R, Gallus A, Ockelford P (2012). Low-dose aspirin for preventing recurrent venous thromboembolism. N Engl J Med.

[63] Haffner SM, Lehto S, Rönnemaa T, Pyörälä K, Laakso M (1998). Mortality from coronary heart disease in subjects with type 2 diabetes and in nondiabetic subjects with and without prior myocardial infarction. N Engl J Med.

[64] Feigin VL, Rinkel GJ, Lawes CM, Algra A, Bennett DA, van Gijn J (2005). Risk factors for subarachnoid hemorrhage: an updated systematic review of epidemiological studies. Stroke.

[65] No authors listed. Aspirin effects on mortality and morbidity in patients with diabetes mellitus. Early treatment diabetic retinopathy study report 14. ETDRS Investigators. JAMA. 1992;268:1292–1300. 10.1001/jama.1992.03490100090033 1507375

[66] No authors listed. Final report on the aspirin component of the ongoing physicians’ health study. Steering Committee of the Physicians’ Health Study Research Group. N Engl J Med. 1989;321:129–135. 10.1056/NEJM198907203210301 2664509

[67] Belch J, MacCuish A, Campbell I, Cobbe S, Taylor R, Prescott R (2008). The prevention of progression of arterial disease and diabetes (POPADAD) trial: factorial randomised placebo controlled trial of aspirin and antioxidants in patients with diabetes and asymptomatic peripheral arterial disease. BMJ.

[68] de Gaetano G, Collaborative Group of the Primary Prevention Project (2001). Low-dose aspirin and vitamin E in people at cardiovascular risk: a randomised trial in general practice. Collaborative group of the primary prevention project. Lancet.

[69] Butalia S, Leung AA, Ghali WA, Rabi DM (2011). Aspirin effect on the incidence of major adverse cardiovascular events in patients with diabetes mellitus: a systematic review and meta-analysis. Cardiovasc Diabetol.

[70] Halvorsen S, Andreotti F, ten Berg JM, Cattaneo M, Coccheri S, Marchioli R (2014). Aspirin therapy in primary cardiovascular disease prevention: a position paper of the European Society of Cardiology working group on thrombosis. J Am Coll Cardiol.

[71] Barnett H, Burrill P, Iheanacho I (2010). Don’t use aspirin for primary prevention of cardiovascular disease. BMJ.

[72] Pearson TA, Blair SN, Daniels SR, Eckel RH, Fair JM, Fortmann SP (2002). AHA guidelines for primary prevention of cardiovascular disease and stroke: 2002 Update: consensus panel guide to comprehensive risk reduction for adult patients without coronary or other atherosclerotic vascular diseases. American Heart Association Science Advisory and Coordinating Committee. Circulation.

[73] Hennekens CH, DeMets DL (2014). Prevention: aspirin in primary prevention needs individual judgements. Nat Rev Cardiol.

[74] Fiscella K, Winters PC, Mendoza M, Noronha GJ, Swanger CM, Bisognano JD (2015). Do clinicians recommend aspirin to patients for primary prevention of cardiovascular disease?. J Gen Intern Med.

[75] Persell SD, Lloyd-Jones DM, Friesema EM, Cooper AJ, Baker DW (2013). Electronic health record-based patient identification and individualized mailed outreach for primary cardiovascular disease prevention: a cluster randomized trial. J Gen Intern Med.

[76] Ghorob A, Bodenheimer T (2012). Sharing the care to improve access to primary care. N Engl J Med.

[77] Sheridan SL, Viera AJ, Krantz MJ, Ice CL, Steinman LE, Peters KE (2010). The effect of giving global coronary risk information to adults: a systematic review. Arch Intern Med.

[78] Pignone M, Alberts MJ, Colwell JA, Cushman M, Inzucchi SE, Mukherjee D (2010). Aspirin for primary prevention of cardiovascular events in people with diabetes: a position statement of the American Diabetes Association, a scientific statement of the American Heart Association, and an expert consensus document of the American College of Cardiology Foundation. Circulation.

[79] Meschia JF, Bushnell C, Boden-Albala B, Braun LT, Bravata DM, Chaturvedi S (2014). Guidelines for the primary prevention of stroke: a statement for healthcare professionals from the American Heart Association/American Stroke Association. Stroke.

[80] Alonso-Coello P, Bellmunt S, McGorrian C, Anand SS, Guzman R, Criqui MH (2012). Antithrombotic therapy in peripheral artery disease: antithrombotic therapy and prevention of thrombosis, 9th ed: American College of Chest Physicians Evidence-Based Clinical Practice Guidelines. Chest.

[81] Bell AD, Roussin A, Cartier R, Chan WS, Douketis JD, Gupta A (2011). The use of antiplatelet therapy in the outpatient setting: Canadian cardiovascular society guidelines executive summary. Can J Cardiol.

[82] Perk J, De Backer G, Gohlke H, Graham I, Reiner Z, Verschuren M (2012). European guidelines on cardiovascular disease prevention in clinical practice (version 2012). The fifth joint task force of the european society of cardiology and other societies on cardiovascular disease prevention in clinical practice (constituted by representatives of nine societies and by invited experts). Eur Heart J.

